# Radiologic Response Combined with Dermatologic Toxicities is the Most Robust Predictor of Survival Benefits in Patients with Inoperable Hepatocellular Carcinoma After Transarterial Chemoembolization Plus Sorafenib Therapy

**DOI:** 10.1007/s00270-021-02846-w

**Published:** 2021-05-04

**Authors:** Zhiqiu Ye, Zhizhen Deng, Suxiang Jiang, Tang Wang, Long Liu, Kuiming Jiang, Yingqiang Zhang

**Affiliations:** 1 Department of Radiology, Guangdong Women & Children Hospital, 520 Xingnan Avenue, Panyu District, Guangzhou, 511400 P.R. China; 2 Department of Obstetrical, Guangdong Women & Children Hospital, Guangzhou, China; 3 Department of Radiology, The Seventh Affiliated Hospital, Sun Yat-Sen University, 628 Zhenyuan Road, Shenzhen, P.R. China

**Keywords:** Hepatocellular carcinoma, Transarterial chemoembolization, Sorafenib, Survival, Predictor

## Abstract

**Purpose:**

The survival benefits of patients with inoperable hepatocellular carcinoma (HCC) who undergo transarterial chemoembolization (TACE) and receive sorafenib therapy remain controversial. We aimed to identify clinical predictors in patients with inoperable HCC undergoing TACE and receiving sorafenib.

**Methods:**

Between January 2014 and December 2017, 148 consecutive patients with inoperable HCC who were treated with TACE plus sorafenib were retrospectively analyzed. Critical clinical factors associated with overall survival (OS) were identified by Cox regression model analysis. Kaplan–Meier methods were used to calculate the survival times, which were compared with the log-rank test.

**Results:**

Macrovascular invasion (MVI), radiologic response and sorafenib-related dermatologic toxicities were identified as independent factors associated with OS. MVI is a known prognostic factor before treatment. The median OS of patients with either radiologic response or dermatologic toxicities was significantly improved compared with that of patients without it (both 23.0 vs. 7.0 months, *P* < 0.001). The median OS of patients with a combination of radiologic response and dermatologic toxicities was significantly longer than that of patients with either radiologic response or dermatologic toxicities, as well as no response (25.0 vs. 14.0 vs. 6.0 months, respectively, *P* < 0.001), and the predictive value was confirmed across patients with different baseline characteristics in terms of MVI, α-fetoprotein level, performance status and liver function.

**Conclusion:**

The combination of radiologic response and sorafenib-related dermatologic toxicities is the most robust predictor of survival benefits for HCC patients after TACE plus sorafenib therapy.

**Level of Evidence:**

Level 3.

**Supplementary Information:**

The online version contains supplementary material available at 10.1007/s00270-021-02846-w.

## Introduction

Approximately half of hepatocellular carcinoma (HCC) cases are diagnosed at an inoperable stage. The treatment options for inoperable HCC are limited, and the prognosis is dismal [1–3]. Based on the evidence of current HCC guidelines, transarterial chemoembolization (TACE) is a common and valuable local therapy for inoperable HCC [1–3]. However, the long-term efficacy of TACE alone remains unsatisfactory due to large tumor load or macrovascular invasion (MVI) [4, 5]. Sorafenib, an oral multikinase inhibitor, is the first approved targeted agent for advanced HCC; it targets vascular endothelial growth factor receptor (VEGFR)-1, 2 and 3, platelet-derived growth factor receptor (PDGFR)-α and β, and serine-threonine kinases (Raf-1 and B-Raf) involved in tumor cell proliferation and tumor angiogenesis [6, 7]. The toxicities related to sorafenib include fatigue, weight loss, diarrhea, anorexia, nausea, vomiting, and hypertension, as well as dermatologic toxicities including rash, desquamation, pruritus, erythema, alopecia, and hand-foot skin reaction (HFSR) [8]. HFSR is a cutaneous reaction characterized by erythema, numbness, tingling, and dysesthesia or paresthesia, particularly on the palms and soles. Some severe cases may present edema, peeling, bleeding, blistering, or ulceration [9].

Previously, several authors have suggested that TACE plus sorafenib may be a “good combination” for inoperable HCC [10, 11]. Several observational studies have demonstrated that TACE plus sorafenib produces additional survival benefits compared with TACE or sorafenib monotherapy for patients with inoperable HCC [12–15]. However, the STAH trial showed no overall survival (OS) benefits in patients with advanced HCC who underwent TACE plus sorafenib compared with those who received sorafenib alone (OS 12.8 months vs. 10.8 months, *P* = 0.290) [16]. Conversely, a large nationwide cohort study in Taiwan showed that TACE plus sorafenib improved survival benefits compared with sorafenib alone in advanced HCC (OS 381 days vs. 204 days, *P* = 0.021) [17]. Additionally, the TACE2 trial showed that drug-eluting beads (DEB-TACE) combined with sorafenib did not improve the progression-free survival (PFS) compared with DEB-TACE plus placebo (PFS 238 days vs. 235 days, *P* = 0.94) in European patients with unresectable HCC [18]; while the TACTICS trial showed that TACE plus sorafenib significantly improve the PFS compared with TACE alone in Asian patients with unresectable HCC (PFS 25.2 months vs. 13.5 months, *P* = 0.006) [19]. Thus, the survival benefits from TACE plus sorafenib remain controversial, and the predictors associated with survival benefits remain unclear.

To date, there have been no definite clinical factors or plasma biomarkers before treatment that can predict survival benefits, and only prognostic factors have been identified, such as MVI, performance status, and alpha-fetoprotein level, which are well-known prognostic factors for HCC patients regardless of whether they receive local regional or systemic therapies [12, 14, 20]. Thus, these prognostic factors may have little clinical value for identifying the survival benefits of HCC patients who receive TACE plus sorafenib. Therefore, this study aimed to: (a) investigate the significant predictors of survival benefits in a cohort of inoperable HCC patients after TACE plus sorafenib therapy, and (b) identify the robust predictors of survival for these patients.

## Materials and Methods

### Study Population

From January 2014 to December 2017, the electronic medical records of HCC patients who received TACE plus sorafenib in our center were retrospectively reviewed. The protocol was approved by the ethics committee of the institutional review board.

The inclusion criteria were as follows: (a) diagnosis of inoperable HCC according to pathology or radiology results (contrast-enhanced computed tomography [CT] or magnetic resonance imaging [MRI]) based on the Chinese guideline for Liver Cancer [3]; (b) macrovascular invasion diagnosed on images, presented as an HCC tumor invading the adjacent portal vein or hepatic vein with a low-attenuation intraluminal venous mass [21, 22]; (c) age 18–75 years; (d) Child–Pugh score less than 8; and (e) Eastern Cooperative Oncology Group (ECOG) score less than 2.

The exclusion criteria were as follows: (a) patients with main portal vein invasion; (b) patients in whom the duration of sorafenib treatment was less than 1 month; (c) patients with serious diseases, for example, jaundice, massive ascites, or other heart or pulmonary diseases; (d) patients with a previous malignancy; and (e) patients who previously received liver surgery or other treatments for HCC.

### Treatment

The treatment protocol was performed as previously described [21, 23]. First, using the Seldinger technique to puncture the femoral artery, a 5 Fr catheter was introduced to the celiac trunk and superior mesenteric artery, and angiography was performed to evaluate the tumor blood supply and to assess the flow of the portal vein. Then, superselective catheterization was performed using a microcatheter to the tumor feeding artery. Next, an emulsion was created using 10–20 mL of Lipiodol (Guerbet, Paris, France) mixed with 20–40 mg of epirubicin (Pfizer, New York, USA). Based on the tumor load, the emulsion was injected into the tumor feeding artery via microcatheter. When the emulsion slowed or backflowed, the injection was stopped, with a maximum volume of no more than 20 mL. Finally, embolization was performed using gelfoam particles (350–560 μm).

Sorafenib was administered on days 1–3 after the TACE procedure. The initial dose was 400 mg twice daily. Based on the grade of toxicity, the dosages were modified. If the patients experienced intolerable toxicities, a standard dosage management process was performed: from 600 mg/d, to 400 mg/d, and then to 400 mg every other day (q.o.d). Patients were encouraged to continue sorafenib treatment until untreatable progression or intolerable toxicities occurred.

### Follow-up

All patients underwent liver contrast-enhanced CT or MRI and laboratory tests to stage the HCC. The laboratory tests included a liver function test, routine blood examination, coagulation function, serum tumor biomarker assay, and hepatitis serologic test, and liver function was recorded as the Child–Pugh score. To evaluate the treatment response, all examinations were repeated 1 month after TACE. TACE treatment was followed by an “on-demand strategy” [23]. For patients without clinical deterioration (ECOG > 2) or liver function deterioration, repeated TACE was indicated when residual viable tumors or new tumors were evident on contrast-enhanced CT or MRI images. For patients who achieved a complete response (CR), follow-up was performed every 2 months and included the above evaluations. OS was documented as the time from the start of treatment until death or the last follow-up.

### Assessment

Tumor response was assessed based on the “primary index tumor response” according to the mRECIST criteria [24]. The primary index tumor was defined as the largest tumor or relevant tumor with macrovascular invasion [25]. The initial response, which was defined with a radiologic assessment at one-month post-TACE, was determined to assess tumor response in the present study [26–28]. All tumor responses were assessed by an independent radiologist and classified as CR, partial response (PR), stable disease (SD) or progressive disease (PD). Whenever the response classification was unclear, a senior radiologist made the final decision. Radiologic response was defined as the sum of CR or PR, while radiologic nonresponse was defined as the sum of SD or PD.

Sorafenib-related toxicities were recorded every 2 weeks after sorafenib therapy via photo or outpatient visits. Prior to therapy, every patient underwent a routine physical examination of the chest, abdomen, head, neck, arms, legs, hands and feet, to rule out skin disease. Dermatologic toxicities were defined as the presentation of skin changes (e.g., grade ≥ 2) within the first month of sorafenib therapy. Toxicities were evaluated by an attending physician (with at least 5 years of experience in oncology) according to the Common Terminology Criteria for Adverse Events (CTCAE) version 4.0. The grade of dermatologic toxicities is listed in Appendix Table 1.

**Table 1:** Baseline patient characteristics

Characteristics	Number (%)
Age (years)*	51.6 ± 10.6
Sex	
Male	143 (96.6)
Female	5 (3.4)
Etiology	
Hepatitis B	142 (95.9)
Hepatitis C	1 (0.7)
Other	5 (3.4)
Cirrhosis	
Present	120 (81.1)
Absent	28 (18.9)
Number of tumors	
1–5	75 (50.7)
> 5	73 (49.3)
Size of main tumor (cm)	
≤ 5	19 (12.8)
5–10	56 (37.8)
> 10	73 (49.3)
ECOG score	
0	25 (16.9)
1	116 (78.4)
2	7 (4.7)
Child–Pugh class	
A	139 (93.9)
B	9 (6.1)
AFP level (ng/mL)	
< 400	44 (29.7)
≥ 400	104 (70.3)
Extrahepatic spread	
Present	8 (5.4)
Absent	140 (94.6)
MVI	
Present	81 (54.7)
Absent	67 (45.3)
Radiological response**	
Responders	68 (45.9)
Nonresponders	80 (54.1)
Dermatologic toxicities	
Present	76 (51.4)
Absent	72 (48.6)

Data are presented as *n* (%). *Mean ± standard deviation. **Responder indicates complete response or partial response; nonresponder indicates stable disease or progressive disease. AFP, alpha-fetoprotein; ECOG, Eastern Cooperative Oncology Group; MVI, macrovascular invasion

### Statistical Analysis

For the baseline characteristics, continuous variables are described as the mean ± standard deviation, and categorical variables are expressed as the number and percentage of patients. OS was calculated by the Kaplan–Meier method and compared using the log-rank test. A univariate analysis was used for all studied variables, and the results were compared using the log-rank test. Variables with *P* < 0.1 in the univariate analysis were entered into a multivariate Cox analysis, which was used to identify risk factors that independently affected OS. All statistical tests were two-sided, and *P* < 0.05 was considered statistically significant. All statistical analyses were performed with SPSS software (SPSS version 16.0, Chicago, IL, USA).

## Results

### Demographics

**Fig. 1:**
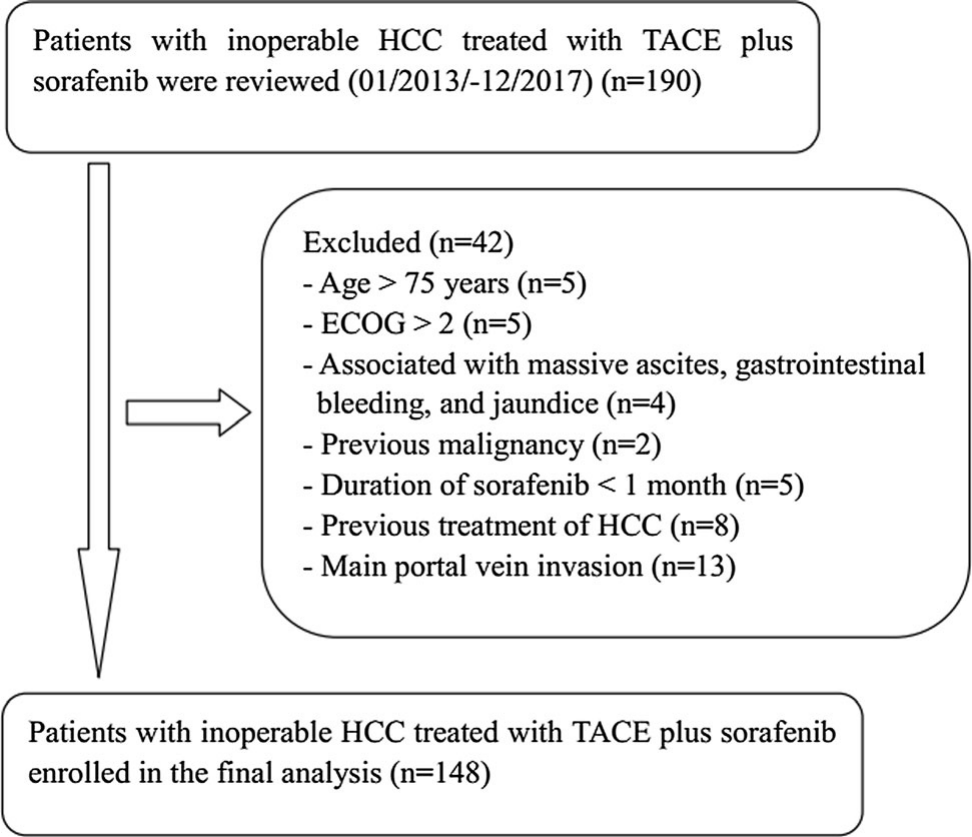
Flow diagram of patient selection. HCC, hepatocellular carcinoma; ECOG, Eastern Cooperative Oncology Group; TACE, transarterial chemoembolization

A total of 148 consecutive patients with inoperable HCC treated with TACE plus sorafenib during the study period were considered in the analysis (Fig. 1). The demographics of the cohort are shown in Table 1. Briefly, the mean tumor size among patients was 8.2 cm. Hepatitis B and liver cirrhosis were the most common diseases. Forty-five percent (*n* = 67) of the patients had Barcelona Clinic Liver Cancer (BCLC) stage B cancer, while the remaining 55% (*n* = 81) of patients had BCLC stage C cancer; among these, 81 showed MVI and 8 showed extrahepatic spread.

### Treatment Outcome

The mean number of TACE sessions per patient was 3.5 (range 2–6). The median duration of sorafenib therapy was 8 months (range 1–50 months). At 1 month post-treatment, the imaging data were assessed for all patients: fourteen patients (9.5%) had CR, 54 (36.5%) had PR, 16 (10.8%) had SD, and 64 (43.2%) had PD. Accordingly, the radiologic response rate was 45.9%. No patients in the present series underwent subsequent liver surgery. Additionally, a total of 105 patients (70.9%) experienced sorafenib-related toxicities within the first month of sorafenib treatment, and the most frequent toxicities were hand-foot-skin reactions, alopecia, weight loss and diarrhea. Among these patients, 76 (51.4%) were documented as having dermatologic toxicities. The most frequent complication related to TACE was post-embolization syndrome. A detailed list of the side effects related to the combination therapy is shown in appendix Table 2.

**Table 2:** Univariate and multivariate analysis of prognostic factors

Variable	Univariate analysis			Multivariate analysis		
	Median OS (mos)	95% CI	*P* value	Hazard ratio	95% CI	*P* value
Age (years)			0.088			0.236
< 52	9.0	5.9, 12.1		0.803	0.560, 1.154	
≥ 52	18.0	13.3, 22.7		1.000		
Number of tumors			< 0.001			0.904
≤ 5	19.0	16.7, 21.3		1.025	0.686, 1.532	
> 5	9.0	6.6, 11.4		1.000		
MVI			< 0.001			0.003
Present	18.0	10.5, 25.5		1.000		
Absent	8.0	6.6, 9.4		1.989	1.255, 3.151	
AFP level (ng/mL)		0.005			0.457	
< 400	23.0	18.9, 27.1		0.847	0.547, 1.312	
≥ 400	10.0	7.7, 12.4		1.000		
ECOG score			0.001			0.218
0–1	14.0	11.1, 16.9		1.000		
2	7.0	4.4, 9.6		1.654	0.743, 3.685	
Radiologic response*			< 0.001			< 0.001
Responders	23.0	19.7, 26.3		1.000		
Nonresponders	7.0	5.8, 8.2		3.549	2.136, 5.898	
Dermatologic toxicities			< 0.001			< 0.001
Present	23.0	20.2, 25.8		1.000		
Absent	7.0	6.1, 7.9		8.074	4.752, 13.7185	

^*^Responders indicates complete response or partial response; nonresponders indicates stable disease or progressive disease. *AFP* alpha-fetoprotein, *ECOG* Eastern Cooperative Oncology Group, *MVI* macrovascular invasion, *CI* confidence interval, *OS* overall survival

### Survival Analysis

Overall, 138 patients (93.2%) died and were censored from follow-up. The median follow-up for these patients was 17.9 months (range 3–63). The median OS for the overall cohort was 13.0 months (95% CI 10.2, 15.8).

In the univariate analysis, the following variables were significantly associated with OS: number of tumors, α-fetoprotein level, ECOG performance status, MVI, radiologic response and dermatologic toxicities. The multivariate analysis demonstrated that MVI, dermatologic toxicities and radiologic response were independent factors for OS (Table 2).

**Fig. 2:**
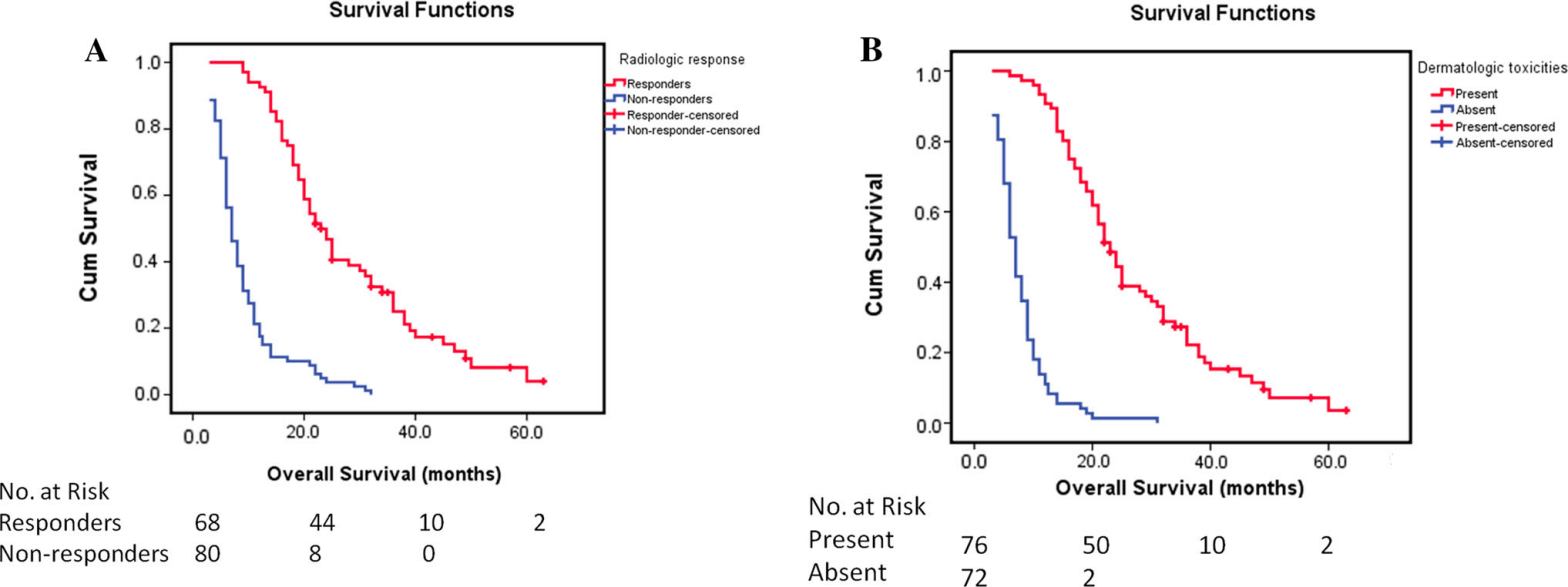
Kaplan–Meier curves showing overall survival (OS) for patients with inoperable hepatocellular carcinoma treated with sorafenib plus transarterial chemoembolization. **A** The median OS for patients with and without a radiologic response was 23.0 months and 7.0 months, respectively (*P* < 0.001). **B** The median OS for patients with and without the presence of dermatologic toxicities was 23.0 months and 7.0 months, respectively (*P* < 0.001)

MVI is a well-known prognostic factor for poor prognosis, and the median OS in this study was 18.0 and 8.0 months for patients without and with MVI, respectively (*P* < 0.001). The median OS was 23.0 months for patients with a radiologic response and 7.0 months for patients without a radiologic response (*P* < 0.001) (Fig. 2A). The median OS was 23.0 and 7.0 months for patients with and without dermatologic toxicities (*P* < 0.001), respectively (Fig. 2B). The median OS values for patients with a radiologic response and for patients with dermatologic toxicities were comparable.

### Survival Stratification

**Fig. 3:**
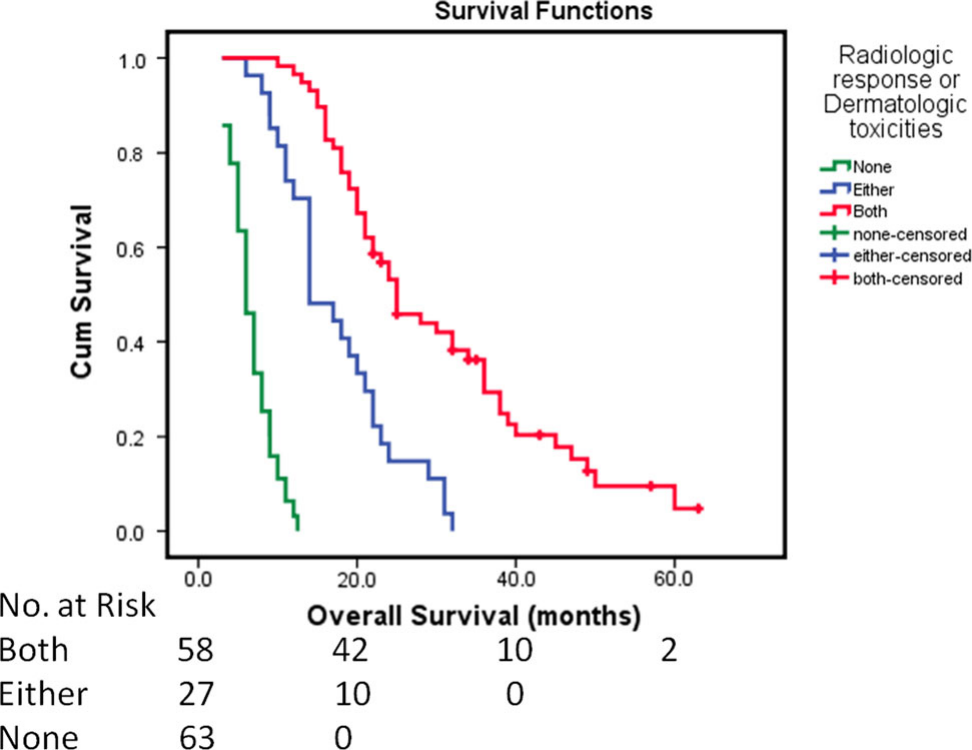
Kaplan–Meier curves showing overall survival (OS) for patients with both radiologic response and dermatologic toxicities, either radiologic response or dermatologic toxicities, and no response after sorafenib plus transarterial chemoembolization. The median OS was 25.0 months, 14.0 months, and 6.0 months, respectively (*P* < 0.001)

Based on the results of this study, radiologic response and dermatologic toxicities were critical predictors of a favorable prognosis. Therefore, three groups were identified based on radiologic response and dermatologic toxicities: patients with both radiologic response and dermatologic toxicities, patients with either radiologic response or dermatologic toxicities, and patients without radiologic response or dermatologic toxicities (hereafter referred to as group A, group B, and group C, respectively). The median survival in the overall cohort for group A, B and C was 25.0, 14.0, and 6.0 months, respectively (*P* < 0.001) (Fig. 3). To validate the predictive value of the combination of radiologic response and dermatologic toxicities across the overall cohort, a subgroup survival analysis of the relevant pretreatment baseline characteristics was conducted, which included MVI, α-fetoprotein level, Child–Pugh class, and ECOG performance status.

**Fig. 4:**
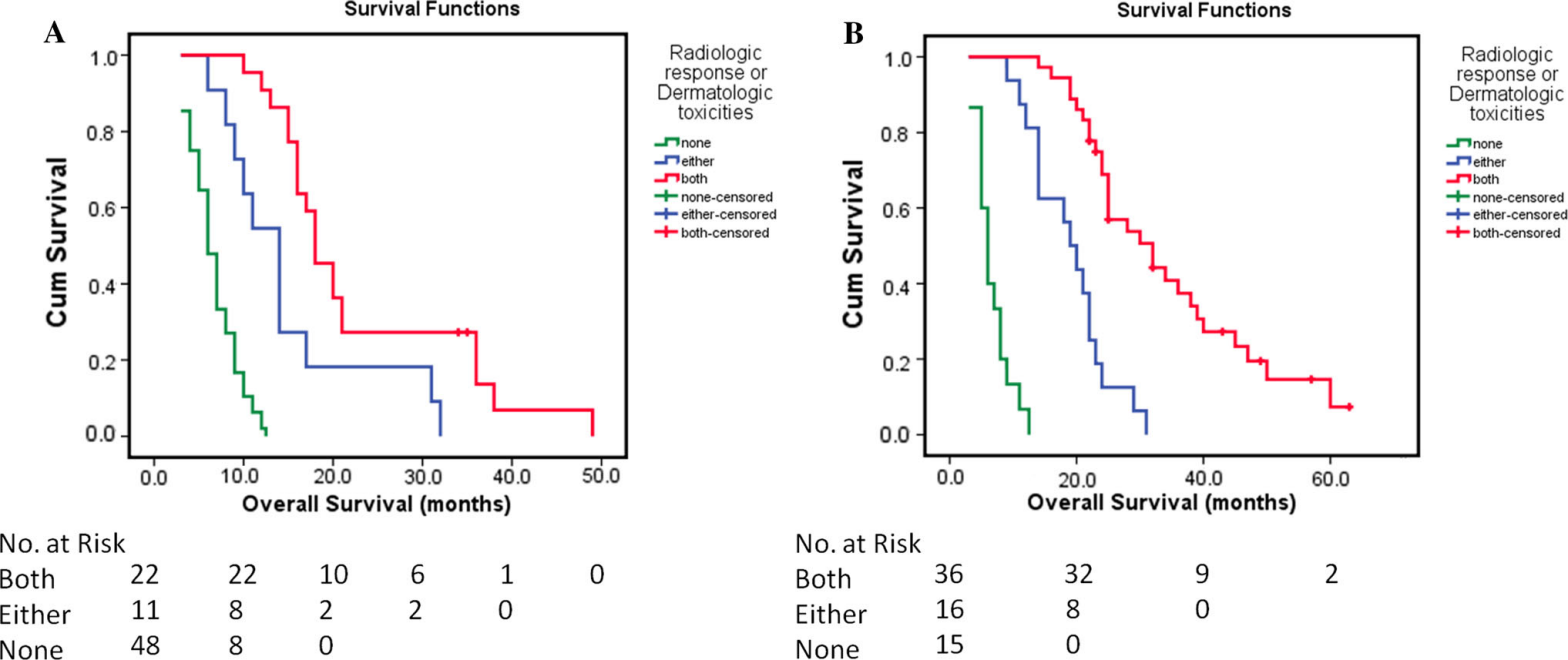
Response-based stratification of the survival of patients with or without presentation of macrovascular invasion (MVI). **A** The median survival values for patients with MVI in the groups with both radiologic response and dermatologic toxicities, either radiologic response or dermatologic toxicities, and no response were 18.0 months, 14.0 months, and 6.0 months, respectively (*P* < 0.001). **B** The median OS for patients without MVI in the groups with both radiologic response and dermatologic responses, either radiologic response or dermatologic toxicities, and no response was 32.0 months, 19.0 months, and 6.0 months, respectively (*P* < 0.001)

The median OS for patients with MVI in group A, B, and C was 18.0, 14.0, and 6.0 months, respectively (*P* < 0.001). The median OS for patients without MVI in group A, B, and C was 32.0, 19.0, and 6.0 months, respectively (*P* < 0.001) (Fig. 4A, B).

The median OS for patients with α-fetoprotein ≤ 400 ng/mL in group A, B, and C was 30.0, 22.0, and 5.0 months, respectively (*P* < 0.001). The median OS for patients with α-fetoprotein > 400 ng/mL in group A, B, and C was 21.0, 14.0, and 6.0 months, respectively (*P* < 0.001).

The median OS for patients with Child–Pugh class A in group A, B, and C was 25.0, 18.0, and 6.0 months, respectively (*P* < 0.001). Because Child–Pugh class B included only 9 patients, a survival analysis was not performed.

The median OS for patients with an ECOG score of 0 in group A, B, and C was 50.0, 20.0, and 6.0 months, respectively (*P* < 0.001). The median OS for patient with an ECOG score of 1 in group with A, B, and C was 21.0, 14.0, and 6.0 months, respectively (*P* < 0.001).

## Discussion

The present study focused on inoperable HCC patients and showed a median survival of 13.0 months, which is comparable to that reported in previous studies of patients with advanced HCC who received sorafenib plus TACE therapy [16, 17, 20]. Overall, the survival rate was better than that in the Asia–Pacific trial, in which patients received sorafenib monotherapy (median OS 6.5 months) [7]. In terms of clinical impact, this study mainly aimed to identify robust predictors of favorable survival. After the final Cox analysis, radiologic response and dermatologic toxicities were found to be independent predictors of survival in patients with inoperable HCC treated with TACE plus sorafenib.

Radiologic assessment plays a critical role in evaluating treatment efficacy and predicting the survival of HCC patients [23–25]. Currently, tumor response assessment mainly includes an evaluation of the initial response and the best response. The best response was defined as the best radiologic response across all time points during repeated “on-demand” sessions. Both the initial response and the best response predict OS well [26]. However, the optimal number of TACE sessions needed to reach the best response in an individual remains unpredictable; consequently, there has been controversy regarding the best time point to evaluate the treatment response after each TACE session for an accurate prediction of OS [28]. Another merit of the initial response is the high follow-up rate. Several studies have demonstrated that the initial radiologic response is a robust predictor of survival [26, 27], which was confirmed by our results.

Sorafenib-related dermatologic toxicities are an important predictor of the treatment response and survival of patients with HCC who undergo sorafenib therapy [9]. Several studies have indicated that dermatologic toxicities are an early biomarker for the prognosis of patients with HCC treated with sorafenib [29–31]. However, the underlying relationship between dermatologic toxicities and treatment response remains unclear, and there are no definite plasma biomarkers that predict these responses [32]. In the present study, the median OS of patients with dermatologic toxicities was significantly higher than that of patients without dermatologic toxicities, which was in line with previous results.

In this study, we attempted to identify the most robust predictor of survival in patients with inoperable HCC receiving TACE plus sorafenib. After the final survival analysis, we found that the combination of radiologic response and dermatologic toxicities was a better predictor of favorable prognosis than either radiologic response or dermatologic toxicities alone, and the predictive value was confirmed across subgroups of patients with different baseline characteristics in terms of MVI, α-fetoprotein level, performance status and liver function. Thus, we believe that the presence of both radiologic response and dermatologic toxicities is the most robust predictor associated with the response to TACE plus sorafenib. A likely explanation is that a radiologic response mainly reflects the efficacy of TACE, while dermatologic toxicities mainly reflect the response to sorafenib. Therefore, the combined presence of radiologic response and dermatologic toxicities predicts the greatest survival benefits for HCC patients who receive TACE plus sorafenib. Recently, a prognostic score model indicated that performance status, portal vein tumor thrombus, radiologic response and dermatologic toxicity could accurately predict survival for patients with HCC undergoing sorafenib plus TACE, and these findings were confirmed by our results [31]. Therefore, based on our results, we believe that inoperable HCC patients, regardless of the presence of MVI or high α-fetoprotein level, may achieve survival benefits after TACE plus sorafenib. Unfortunately, there were no definite factors to predict the response before treatment, especially regarding sorafenib-related dermatologic toxicities. Thus, the discordance of results regarding survival benefits between randomized control trials (for example, the STAH and TACE2 trials) [16, 18] and retrospective observational studies [12–15, 17] may be addressed using our results. It is known that retrospective observational studies have potential selection bias (i.e., patients with longer survival times may have a longer duration of sorafenib treatment).

This study has some limitations. First, it is a retrospective study with a relatively small sample size. Second, although sorafenib-related dermatologic toxicities were identified as a robust predictor of survival benefits, no definitive factors were identified to accurately predict the response before treatment. Nevertheless, this predictor may be helpful for identifying whether a patient might benefit from sorafenib and for decision-making regarding whether to continue therapy after the first cycle of treatment.

In conclusion, the results of this study suggest that radiologic response and sorafenib-related dermatologic toxicities are critical predictors of favorable survival in HCC patients after TACE plus sorafenib. The combined presence of both radiologic response and dermatologic toxicities performed better than the presence of radiologic response or dermatologic toxicities alone in predicting the survival benefits for these patients.

## Supplementary Information

Below is the link to the electronic supplementary material.

Supplementary file1 (DOC 92 kb)
