# Supplementary material for: Radiologic Response Combined with Dermatologic Toxicities is the Most Robust Predictor of Survival Benefits in Patients with Inoperable Hepatocellular Carcinoma After Transarterial Chemoembolization Plus Sorafenib Therapy
Source: Cardiovasc Intervent Radiol. 2021 May 4;44(9):1394–402. doi: 10.1007/s00270-021-02846-w (PMC8382652; doi:10.1007/s00270-021-02846-w)
Supplement: Supplementary file 1 — Supplementary file1 (DOC 92 kb) [file 270_2021_2846_MOESM1_ESM.doc]

Appendix Table 1: Definition and/or grading of sorafenib-related dermatological toxicities

| Grade | Definition |
| --- | --- |
| Grade 0 | None |
| Grade 1 | Numbness |
|  | Unpleasant sensation when touching ordinary things |
|  | Burning or prickling feeling |
|  | Erythema |
|  | Tingling |
|  | Painless swelling |
|  | Redness or discomfort of hands or feet |
| Grade 2 | One or more of the following: |
|  | Painful redness |
|  | Swelling |
|  | Skin thickening of the hands or feet |
|  | Symptoms that create discomfort but do not affect the patients’s normal daily activities |
| Grade 3 | One or more of the following: |
|  | Scaling or shedding of skin |
|  | Ulcerative |
|  | Blistering |
|  | Severe pain of the hands and feet |
|  | Skin thickening |
|  | Severe discomfort that causes the patient to be unable to work or perform daily activities. |

Appendix Table 2. Adverse events related to combination therapy of TACE plus sorafenib.

| Adverse Events | Grade 1 | Grade 2 | Grade 3 | Grade 4 |
| --- | --- | --- | --- | --- |
| Abdominal pain | 35(23.6) | 52(35.1) | 0 | 0 |
| Fever (>38.5 °C) | 32(21.6) | 35(23.6) | 1(0.7) | 0 |
| Liver abscess | 0 | 0 | 1(0.7) | 0 |
| Vomiting | 33(22.3) | 30(20.3) | 0 | 0 |
| New ascites | 8(5.4) | 7(4.7) | 2(1.4) | 0 |
| Pleural effusion | 0 | 4(2.7) | 1(0.7) | 0 |
| Liver dysfunction | 3(2.0) | 4(2.7) | 3(2.0) | 0 |
| Inguinal hematoma | 11(7.4) | 0 | 0 | 0 |
| Gastrointestinal bleeding | 0 | 0 | 2(1.4) | 0 |
| Dermatologic toxicities  - HFRS  - Rash  - Alopecia | 25(32.4)  21(14.2)  7(4.7)  48(32.4) | 48(32.4)  39(26.4)  11(7.4)  20(13.5) | 28(18.9)  23(15.5)  8(5.4)  0 | 0 |
| Diarrhea | 10(6.8) | 34(23.0) | 24(16.2) | 0 |
| Hypertension | 4(2.7) | 2(1.4) | 1(0.7) | 0 |
| Weight loss | 27(18.2) | 33(22.2) | 0 | 0 |
| Fatigue | 30(20.3) | 21(14.2) | 0 | 0 |
| Anorexia | 26(17.6) | 18(12.2) | 3(2.0) | 0 |

Data are presented as n (%). TACE, transarterial chemoembolization; HFRS, hand-foot skin reaction.
